# Endoscopic characteristics and performance of WASP classification in the diagnosis of colorectal sessile‐serrated lesions in Vietnamese patients

**DOI:** 10.1002/jgh3.13109

**Published:** 2024-06-24

**Authors:** Nhu Thi Hanh Vu, Huy Minh Le, Diem Thi‐Ngoc Vo, Nhan Quang Le, Dung Dang Quy Ho, Duc Trong Quach

**Affiliations:** ^1^ Department of Internal Medicine University of Medicine and Pharmacy at Ho Chi Minh City Ho Chi Minh Vietnam; ^2^ GI Endoscopy Department University Medical Center Ho Chi Minh City Ho Chi Minh Vietnam; ^3^ Department of Histology‐Embryology and Pathology University of Medicine and Pharmacy at Ho Chi Minh City Ho Chi Minh Vietnam; ^4^ Department of Endoscopy Cho Ray Hospital Ho Chi Minh City Vietnam

**Keywords:** colorectal cancer, endoscopic characteristics, sessile‐serrated lesions, Vietnam

## Abstract

**Background/Aims:**

Sessile‐serrated lesions (SSLs) are challenging to detect due to their typically subtle appearance. The Workgroup serrAted polypS and Polyposis (WASP) classification was developed to diagnose SSLs endoscopically. This study aimed to evaluate the endoscopic characteristics of SSLs and the performance of the WASP classification in the Vietnamese population.

**Methods:**

This cross‐sectional study was carried out on patients with lower gastrointestinal symptoms who underwent colonoscopy at a Vietnamese tertiary hospital. Univariate and multivariate analyses were performed to identify endoscopic features associated with SSLs. The performance of the WASP classification for diagnosing SSLs was assessed, and SSLs were diagnosed according to the 2019 World Health Organization (WHO) criteria.

**Results:**

There were 2489 patients, with a mean age of 52.1 ± 13.1 years and a female‐to‐male ratio of 1:1.1. A total of 121 specimens from 105 patients were diagnosed with SSLs. According to multivariate analysis, the endoscopic features significantly associated with SSLs were proximal location (odds ratio [OR]: 2.351; 95% confidence interval [CI]: 1.475–3.746), size >5 mm (OR: 2.447; 95% CI: 1.551–3.862), flat morphology (OR: 2.781; 95% CI: 1.533–5.044), irregular shape (OR: 4.516; 95% CI: 2.173–9.388), varicose microvascular vessels (OR: 5.030; 95% CI: 2.657–9.522), and dark spots inside the crypts (OR: 5.955; 95% CI: 3.291–10.776). The accuracy of the WASP classification for diagnosing SSLs was 94.0% (95% CI: 92.8%–95.0%).

**Conclusion:**

Proximal location, size >5 mm, flat morphology, irregular shape, varicose microvascular vessels, and dark spots inside the crypts were significantly associated with SSLs. The WASP classification had high accuracy in the diagnosis of SSLs.

## Graphical abstract

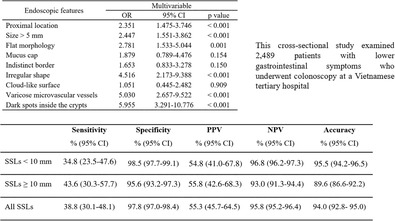

## Introduction

Colorectal cancer (CRC) is still the second leading cause of cancer‐related mortality worldwide.^1^ In Vietnam, CRC ranks fourth among all malignancies in men and second in women, with more than 182 000 new cases diagnosed in 2020.^1^ It is estimated that up to 30% of all CRCs develop from serrated lesions via the serrated neoplasia pathway.^2^ In addition, the serrated pathway carries a greater risk of developing CRC than does the traditional adenoma–carcinoma pathway due to its more aggressive pathophysiology.^3^, ^4^ Sessile‐serrated lesions (SSLs) are the most prevalent and significant precancerous serrated lesions that progress to cancer through cytological dysplasia.^5^, ^6^

Endoscopically, detecting and discriminating SSLs from other benign polyps may be challenging because of their subtle appearance.^7^, ^8^ Furthermore, due to their indistinct borders, these tumors are frequently incompletely resected and consequently associated with interval and synchronous CRC.^9^ Hence, identifying the specific endoscopic characteristics of SSLs may improve SSL detection and diagnostic accuracy, leading to appropriate treatments and ultimately preventing the development of CRC.^10^

Using endoscopic classifications is crucial in clinical practice for optimizing and providing a standardized approach to diagnosing SSLs. Unfortunately, SSLs are often misclassified as non‐neoplastic according to the narrow‐band imaging international colorectal endoscopic (NICE) classification or the Japanese narrow‐band imaging expert team classification.^11^ Therefore, the Workgroup serrAted polypS and Polyposis (WASP) classification was developed by combining the NICE classification with the SSL characterization criteria, allowing the differentiation of hyperplastic polyps (HPs), adenomas, and SSLs.^12^ However, the diagnostic value of the WASP classification has been limited and controversial, especially in Asian countries.

In Vietnam, there is a paucity of evidence on the use of endoscopic features, especially the WASP classification in SSL patients. Therefore, we aimed to evaluate the endoscopic characteristics and performance of the WASP classification for diagnosing SSLs in the Vietnamese population. The data from this study may contribute to more valuable insights into SSLs, and we can tailor our solutions accordingly to improve colonoscopy quality, resulting in a reduction in CRC incidence and mortality rate.

## Methods

### Study participants

A cross‐sectional study was conducted on outpatients who reported lower gastrointestinal symptoms and underwent colonoscopy at the University Medical Centre at Hochiminh City, Vietnam, between March 2022 and July 2023. The exclusion criteria were age under 18 years, history of CRC or colorectal surgery, inherited cancer syndromes, coagulation disorders, inflammatory bowel disease, unqualified bowel preparation determined by the Boston Bowel Preparation Scale (BBPS) with a total score of <6 and a region score of <2,^13^ incomplete colonoscopies, withdrawal time of less than 6 min, and unwillingness to participate. If patients received multiple colonoscopies during the study period, only the first colonoscopy was included in the analysis. Participants with a body mass index equal to or greater than 25.0 kg/m^2^ were defined as obesity.

Written informed consent was obtained from all participants. The study protocol was approved by the Board of Ethics in Biomedical Research of the University of Medicine and Pharmacy at Ho Chi Minh City (ID number: 615/HDDD‐DHYD, signed on November 19, 2021). All clinical investigations were conducted according to the ethical guidelines of the Declaration of Helsinki.

### Colonoscopy procedure

All the subjects received dietary instructions before colonoscopy, and bowel preparation was performed with 3 L of polyethylene glycol‐based material (Fortrans®, Beaufour Ipsen Industrie, France). Colonoscopies were performed by seven experienced endoscopists using the Olympus Evis Exera III High Definition CV‐190 (Olympus Co., Ltd., Tokyo, Japan). All participating endoscopists had an adenoma detection rate of more than 30% and had performed at least 3000 colonoscopic procedures over the last 5 years. Furthermore, prior to the commencement of the study, all the endoscopists had attended the web‐based training program (CATCH project) to identify flat and depressed colorectal lesions.^14^ Moreover, they also attended a local training session to standardize the examination process, identify the characteristics of SSLs, and evaluate the WASP classification. Therefore, the diagnostic ability of the participating endoscopists was assessed after training, and high‐performance colonoscopies were performed.

The morphology, location, and size of all polyps detected were prospectively recorded and analyzed. The participating endoscopists directly evaluated and recorded these characteristics based on the available data collection sheets. The polyp macroscopic type was divided into three categories according to the Paris classification: type 0‐I: polypoid (0‐Is: sessile, 0‐Ip: pedunculated); type 0‐II: nonpolypoid (0‐IIa: slightly elevated, 0‐IIb: flat, 0‐IIc: slightly depressed); and type 0‐III: excavated.^15^ The flat morphology included type 0‐II and type 0‐III. The location of the polyps was categorized as the cecum, ascending, transverse, descending, sigmoid colon, or rectum. The proximal colon included the cecum, ascending colon, hepatic flexure, and transverse colon, while the distal colon included the splenic flexure, descending colon, sigmoid colon, and rectum. The size of the lesions was determined by comparing them with open (with a width of 7 mm) or closed (equal to 3 mm) biopsy forceps or polypectomy snares of known diameters.

Endoscopic characteristics of the SSLs were analyzed using previously validated criteria defined by Hazewinkel et al.^7^ and Murakami et al.^16^ The mucus cap was defined as a focused accumulation of mucus on the mucosal surface (clear, bile‐stained, or debris‐stained) that can be eliminated with irrigation. Indistinct borders were described as vague demarcations of the lesion border. The irregular shape was characterized by an asymmetric shape, unlike the oval, circular shape of small HPs and conventional adenomas. A cloud‐like surface appeared as a soft‐looking nodule or bumpy, resembling the surface of a cumulus cloud. Varicose microvascular vessels were defined as vessels thicker than meshed capillary vessels that meandered similarly to varicose veins. Dark spots inside the crypts were described as small dark dots inside the open crypts.

**Figure 1:**
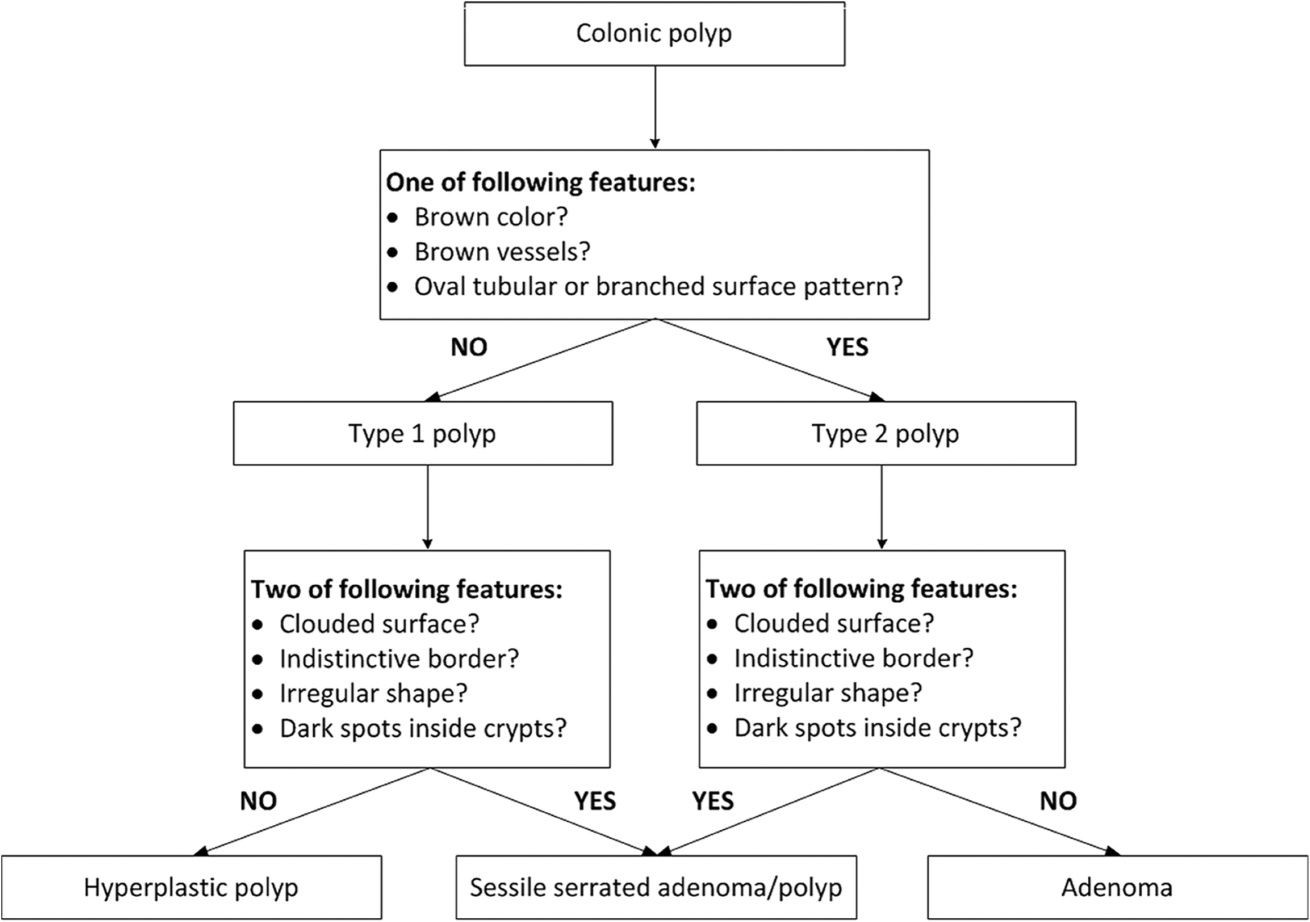
The WASP classification. WASP: Workgroup serrAted polypS and polyp.

The WASP classification was applied for every polyp detected. The polyps were first evaluated using the NICE criteria to distinguish between polyps resembling HPs (type 1 polyps) or adenomas (type 2 polyps).^17^ A type 2 polyp was identified by the presence of at least one of the following adenoma‐like features: (1) a darker color than the surrounding mucosa, (2) visible brown vessels, or (3) an oval, tubular, or branched surface pattern. Subsequently, the diagnostic criteria for SSLs were used to separate SSLs and HPs for type 1 polyps and adenomas for type 2 polyps. The presence of at least two of the following SSL‐like features was sufficient for the diagnosis of SSLs: (1) a cloudy surface, (2) indistinctive borders, (3) irregular shapes, or (4) dark spots inside the crypt^12^ (Fig. 1).

Cecal intubation was confirmed by identifying the appendiceal orifice, cecal valve, or intubation of the ileum. Stopwatches were used to record withdrawal times, which were required at least 6 min after subtracting the time needed for polypectomies. All lesions were removed using standard procedures, as determined by the endoscopist, including biopsy forceps, cold snare, hot polypectomy, endoscopic mucosal resection, and endoscopic submucosal dissection. Non‐pedunculated diminutive lesions ≤3 mm were removed by cold biopsy forceps. Non‐pedunculated lesions >3 mm were resected by cold snare polypectomy. Hot snare polypectomy was used to remove 10‐ to 19‐mm non‐pedunculated and pedunculated lesions ≥10 mm. Large nonpedunculated lesions (≥20 mm) were resected by endoscopic mucosal resection or endoscopic submucosal dissection.

### Histopathological analysis

**Figure 2:**
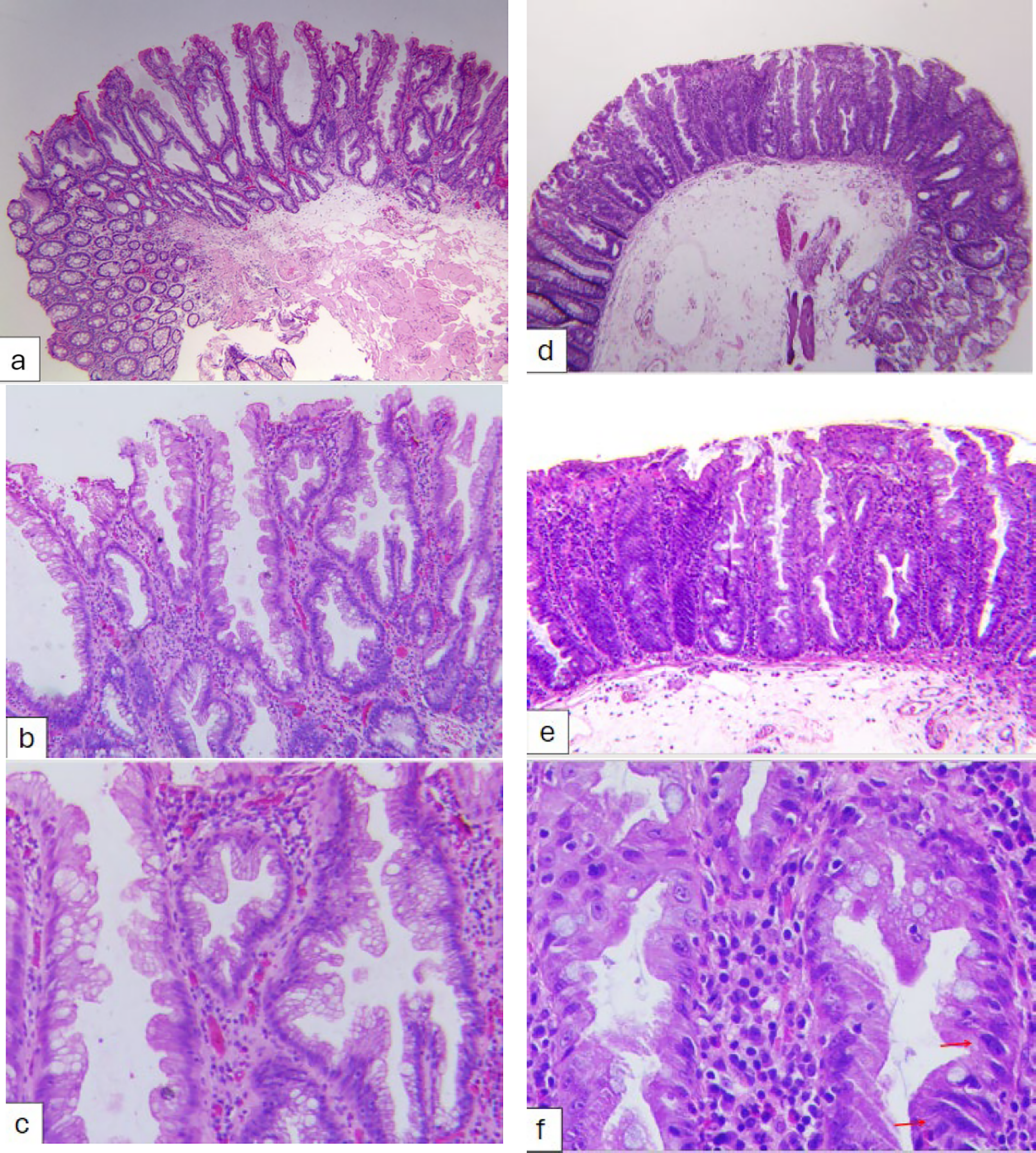
Examples for sessile‐serrated lesion (SSL) and sessile‐serrated lesion with dysplasia (SSLD) with series figures of 4×,10×, and 40×, respectively. SSL (a, b, and c) has bland cytology and crypts with prominent serrations. SSL contains a mixture of goblet cells, cells with microvesicular mucin droplets, overall distortion of the normal crypt architecture, defined as horizontal growth along the muscularis mucosae, dilation of the crypt base, and serrations extending into the crypt base. Nuclear atypia is not defined in this case (c). At low‐to‐medium magnification, this SSLD shows subtle architectural changes with mild crowding of crypts separated by less lamina propria and minimal degree of disorganization (d, e, and f). The cells are hypermucinous with some crowding of nuclei, focal hyperchromasia, and loss of polarity (red arrow, f). These findings were considered as minimal deviation dysplasia of SSLD.

Histopathological diagnosis was used as a reference standard for all lesions. All resected lesions were collected in separate jars and fixed in 10% formalin. Sections were cut at 4 μm thickness and stained with hematoxylin–eosin. Serrated lesions were diagnosed according to the updated 2019 World Health Organization (WHO) criteria.^18^, ^19^, ^20^ A single unequivocally distorted crypt was regarded as a diagnostic criterion for SSL (Fig. 2). The histologic features of these crypts were determined if they had one of the following characteristics: (1) a horizontally growing crypt along the muscularis mucosa (L‐shaped or inverted T‐shaped crypt); (2) dilatation of the crypt base (basal one‐third of the crypt); (3) serrations extending into the crypt base; or (4) asymmetrical proliferation of the crypts. SSLs with dysplasia were further categorized accordingly. The dysplastic part was distinguished from the SSL by architectural changes such as villous architecture, elongated crypts, crowded crypts with complex branching, cribriforming, and altered luminal serration compared with the background SSL.^18^ As there were reports about the low interobserver agreement among pathologists in the differentiation of SSLs from HPs,^21^ all the lesions detected in our study were evaluated by two experienced gastrointestinal pathologists, and any disagreements were resolved by re‐evaluation until a consensus was reached.

### Statistical analysis

All the statistical analyses were performed using SPSS software version 23 (SPSS, Inc., Chicago, IL) and MedCalc® Statistical Software version 19.6.1 (MedCalc Software Ltd., Ostend, Belgium). Continuous variables were assessed for a normal distribution through the Kolmogorov–Smirnov test. Variables conforming to a normal distribution were presented as the mean and standard deviation (SD) and were analyzed using the t test. Variables not normally distributed were presented as medians (including upper and lower quartiles) and were analyzed using the Mann–Whitney *U* test. Categorical variables were expressed as numbers and percentages and were compared using Pearson's chi‐square test. Univariate and multivariate logistic regression analyses were performed to assess endoscopic features associated with SSLs. All *P* values less than 0.05 were considered to indicate statistical significance. The sensitivity, specificity, positive predictive value, negative predictive value, and accuracy of the WASP classification were calculated using histopathological diagnosis as the reference standard.

## Results

### Characteristics of participants

**Figure 3:**
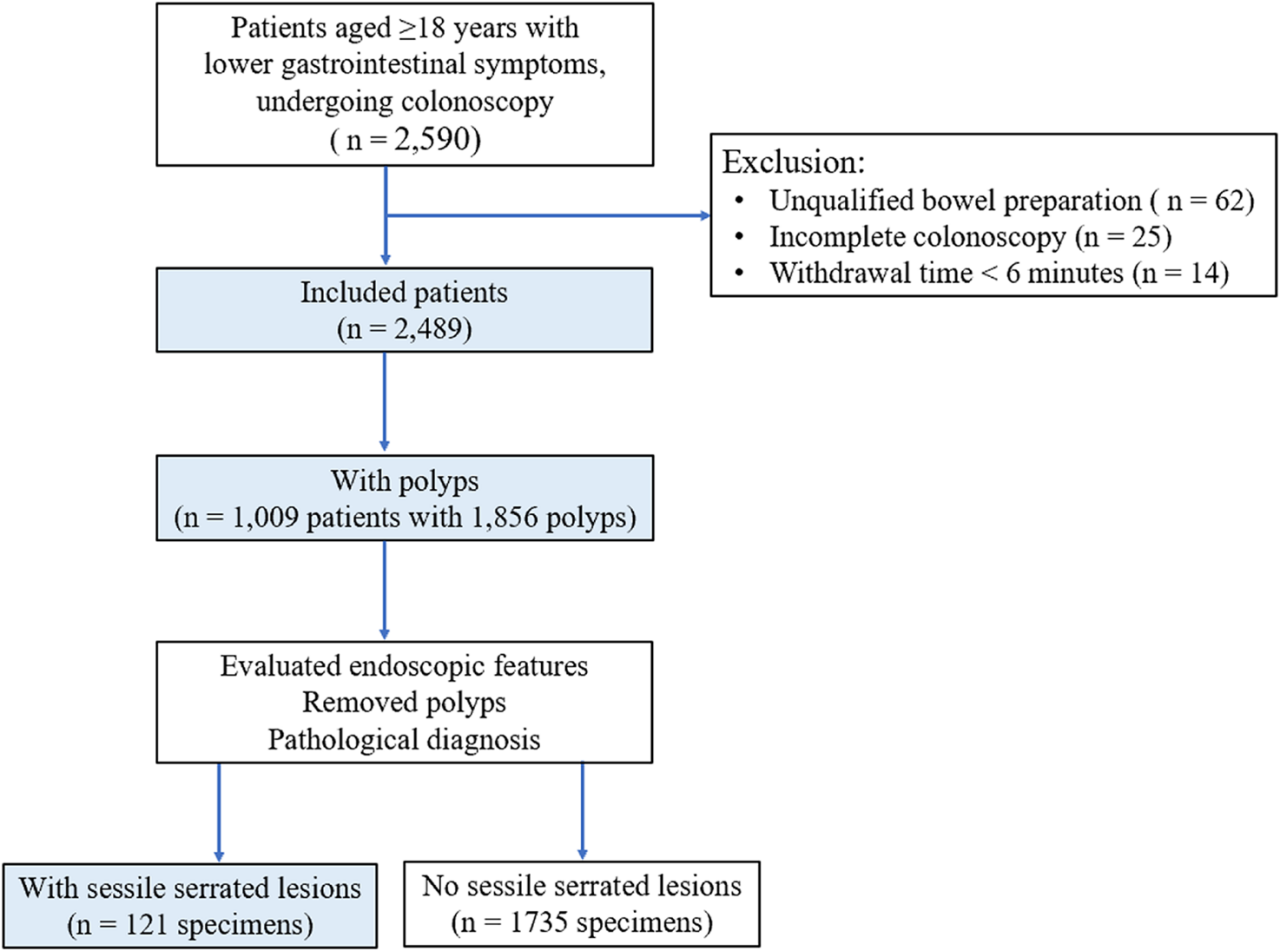
Flowchart of participant enrollment.

A total of 2590 participants with lower gastrointestinal symptoms underwent colonoscopy. We excluded 101 patients due to incomplete colonoscopies, unqualified bowel preparation, or a withdrawal time of less than 6 min. As a result, 2489 patients were included in the study. Among the 1856 lesions removed from 1009 patients, 121 were histopathologically diagnosed as SSLs (Fig. 3). The histopathological diagnoses of all polyps included 1384 (74.6%) adenomas, 214 (11.5%) HPs, 121 (6.5%) SSLs, 20 (1.1%) traditional serrated adenomas, 17 (0.9%) carcinomas, and 100 (5.4%) others. Other polyps included inflammatory polyps, unclassified serrated adenomas, juvenile polyps, and leiomyomas.

The main indications for colonoscopy were abdominal pain (62.4%), diarrhea (51%), constipation (29.2%), and hematochezia (13.8%). The mean age of the patients was 52.1 ± 13.1 years (range: 19–87 years). The female‐to‐male ratio was 1:1.1.

### Demographic and endoscopic characteristics of the SSLs

A total of 121 lesions from 105 patients were histopathologically diagnosed with SSLs. There were 72 specimens of SSLs (59.5%) with dysplasia.

The mean age of the SSL patients was 57.6 ± 12 years, ranging from 21 to 84 years, and 91.4% were older than 40 years. A greater percentage of the patients were male (61%). There was a notable trend in the SSL prevalence with increasing age.

The percentage of patients aged less than 40 years among SSL patients was only 8.6%; moreover, this prevalence increased to 33.3% and 44.8% in those aged 50–59 years and those aged ≥60, respectively. Table 1 shows the demographic characteristics of the SSL patients.

**Table 1:** Demographic characteristics of SSL patients

Characteristics	SSLs, *n* (%)
	*N* = 105
Sex	
Male	64 (61.0)
Female	41 (39.0)
Age	
<40	9 (8.6)
40–49	14 (13.3)
50–59	35 (33.3)
60–69	30 (28.6)
≥70	17 (16.2)
Obesity	
Yes	25 (23.8)
No	80 (76.2)
Smoking	
Yes/ever	38 (36.2)
No	67 (63.8)
Alcohol consumption	
Yes	20 (19.0)
No	85 (81.0)
Hypertension	
Yes	35 (33.3)
No	70 (66.7)
Diabetes mellitus	
Yes	20 (19.0)
No	85 (81.0)
Family history of CRC	
Yes	7 (6.7)
No	98 (93.3)

There were 90 patients (85.7%) with one SSL, and 15 patients had two SSLs (14.3%). The endoscopic findings of the SSLs are summarized in Table 2. The most common locations of SSLs were the ascending colon (38.0%) and transverse colon (19.0%). A total of 70.2% (*n* = 85) of the SSLs were located in the proximal colon, and 20.7% (*n* = 25) of those had a flat morphology. The average size of the SSLs was 8.8 ± 4.8 cm. On NBI, the majority of the SSLs were NICE type 1, accounting for 52.9%.

**Table 2:** Endoscopic characteristics of sessile‐serrated polyps

Characteristics	All polyps (*n*, %)	SSLs (*n*, %)	Non‐SSLs (*n*, %)
	*N* = 1856	*N* = 121	*N* = 1735
Location			
Cecum	112 (6)	16 (13.2)	96 (5.5)
Ascending colon	341 (18.4)	46 (38)	295 (17.0)
Transverse colon	414 (22.3)	23 (19)	391 (22.5)
Descending colon	249 (13.4)	9 (7.4)	240 (13.8)
Sigmoid colon	515 (27.7)	15 (12.4)	500 (28.8)
Rectum	225 (12.1)	12 (9.9)	213 (12.3)
Size (mm)			
≤5	1060 (57.1)	40 (33.1)	1020 (58.8)
6–10	574 (30.9)	55 (45.5)	519 (29.9)
11–20	197 (10.6)	24 (19.8)	173 (10.0)
>20	25 (1.3)	23 (1.3)	2 (1.7)
Shape (Paris classification)			
0‐Is	1483 (79.9)	84 (69.4)	1399 (80.6)
0‐Ip	232 (12.5)	12 (9.9)	220 (12.7)
0‐IIa	137 (7.5)	23 (19)	114 (6.6)
0‐IIb	3 (0.2)	2 (1.7)	1 (0.1)
0‐IIc	1 (0.1)	0 (0)	1 (0.1)
NICE type			
NICE 1	467 (25.2)	64 (52.9)	403 (23.2)
NICE 2	1389 (74.8)	57 (47.1)	1332 (76.8)
NICE 3	0 (0)	0 (0)	0 (0)
Mucus cap			
Yes	42 (2.3)	19 (15.7)	23 (1.3)
No	1814 (97.7)	102 (84.3)	1712 (98.7)
Indistinct border			
Yes	105 (5.7)	38 (31.4)	67 (3.9)
No	1751 (94.3)	63 (68.6)	1668 (96.1)
Irregular shape			
Yes	58 (3.1)	27 (22.3)	31 (1.8)
No	1798 (96.9)	94 (77.7)	1704 (98.2)
Cloud‐like surface			
Yes	50 (2.7)	23 (19)	27 (1.6)
No	1806 (97.3)	98 (81)	1708 (98.4)
Varicose microvascular vessels			
Yes	71 (3.8)	36 (29.8)	35 (2.0)
No	1785 (96.2)	85 (70.2)	1700 (98.0)
Dark spots inside the crypts			
Yes	94 (5.1)	47 (38.8)	47 (2.7)
No	1762 (94.9)	74 (61.2)	1688 (97.3)

SSLs, sessile‐serrated lesions.

**Figure 4:**
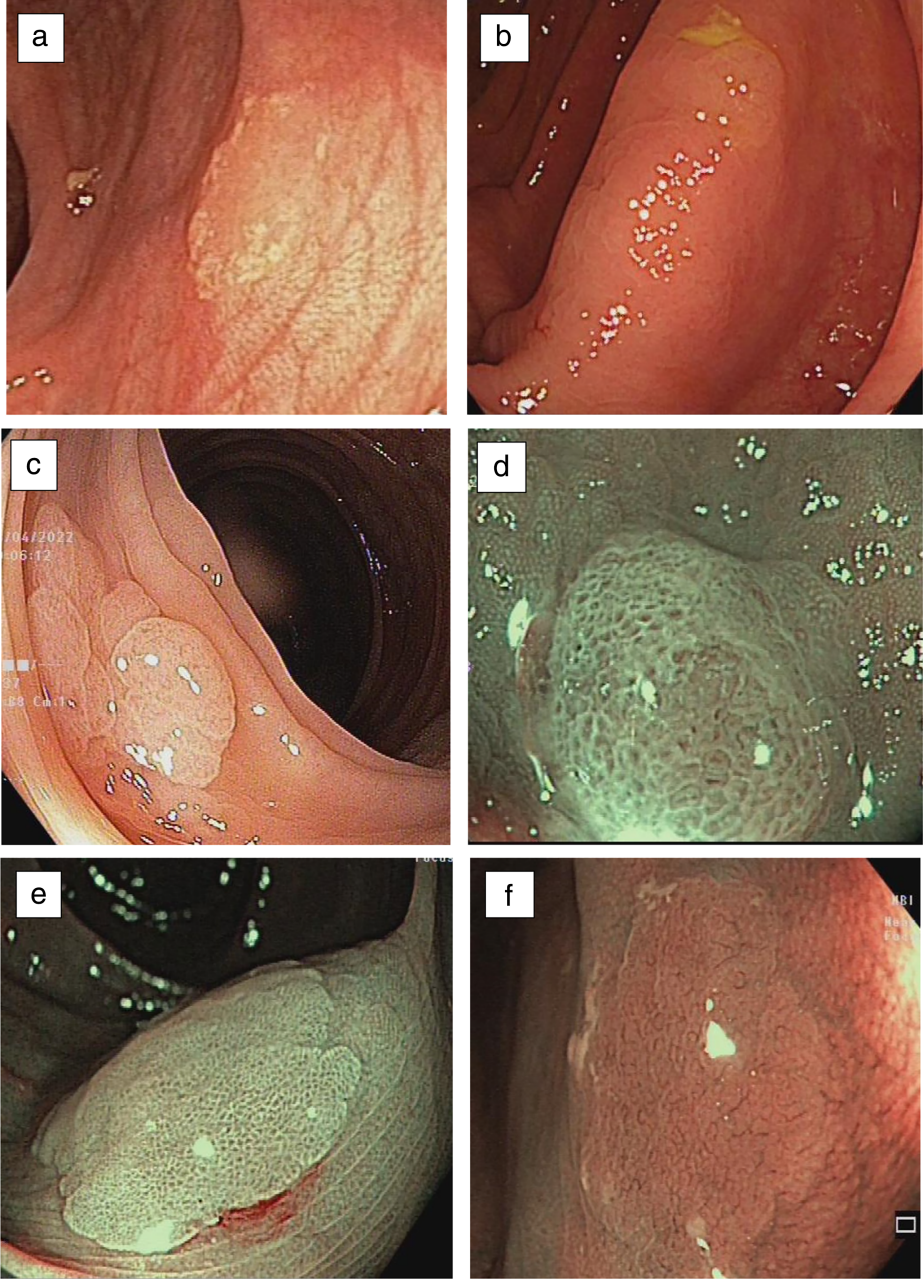
Endoscopic findings of sessile‐serrated lesions. (A) Mucus cap. (A, B, E, F) Flat morphology. (B) Indistinct border. (C) Irregular shape. (D, E) Dark spots inside the open crypts. (F) Varicose microvascular vessels.

Figure 4 shows the endoscopic findings of sessile‐serrated lesions in our study. Dark spots inside the crypts and indistinct borders were the most prevalent endoscopic features of SSLs, with 38.8% and 31.4%, respectively.

Table 3 shows the results of the univariate and multivariate analyses of endoscopic characteristics associated with SSLs. On per‐polyp univariate analysis of endoscopic factors, proximal location, size >5 mm, flat morphology, mucus cap, indistinct borders, irregular shape, cloud‐like surface, varicose microvascular vessels, and dark spots inside the crypts were significantly related to SSLs. According to our multivariate analysis, the factors significantly associated with the SSLs were dark spots inside the crypts (odds ratio [OR]: 5.955; 95% confidence interval [CI]: 3.291–10.776; *P* < 0.001), varicose microvascular vessels (OR: 5.030; 95% CI: 2.657–9.522; *P* < 0.001), irregular shape (OR: 4.516; 95% CI: 2.173–9.388; *P* < 0.001), flat morphology (OR: 2.781; 95% CI: 1.533–5.044; *P* = 0.001), size >5 mm (OR: 2.447; 95% CI: 1.551–3.862; *P* < 0.001), and proximal location (OR: 2.351; 95% CI: 1.475–3.746; *P* < 0.001).

**Table 3:** Univariate and multivariate logistic regression analyses of endoscopic features in sessile‐serrated lesions

Endoscopic features	Univariable			Multivariable		
	OR	95% CI	*P* value	OR	95% CI	*P* value
Proximal location	2.877	1.927–4.297	<0.001	2.351	1.475–3.746	<0.001
Size >5 mm	2.889	1.955–4.269	<0.001	2.447	1.551–3.862	<0.001
Flat morphology	3.635	2.252–5.866	<0.001	2.781	1.533–5.044	0.001
Mucus cap	13.865	7.314–26.285	<0.001	1.879	0.789–4.476	0.154
Indistinct border	11.398	7.232–17.965	<0.001	1.653	0.833–3.278	0.150
Irregular shape	15.789	9.053–27.535	<0.001	4.516	2.173–9.388	<0.001
Cloud‐like surface	14.847	8.211–26.843	<0.001	1.051	0.445–2.482	0.909
Varicose microvascular vessels	20.571	12.307–34.385	<0.001	5.030	2.657–9.522	<0.001
Dark spots inside the crypts	22.811	14.306–36.371	<0.001	5.955	3.291–10.776	<0.001

CI, confidence interval; OR, odds ratio.

### Diagnostic reliability of the WASP classification for sessile‐serrated polyps

The sensitivity, specificity, positive predictive value, negative predictive value, and accuracy of the WASP classification for diagnosing SSLs are described in Table 4. The WASP classification demonstrated superior diagnostic performance in diagnosing SSLs less than 10 mm, with specificity, negative predictive value, and accuracy greater than 95%.

**Table 4:** Diagnostic performance of the WASP classification for differentiating sessile‐serrated lesions

	Sensitivity	Specificity	PPV	NPV	Accuracy
	% (95% CI)	% (95% CI)	% (95% CI)	% (95% CI)	% (95% CI)
SSLs < 10 mm	34.8 (23.5–47.6)	98.5 (97.7–99.1)	54.8 (41.0–67.8)	96.8 (96.2–97.3)	95.5 (94.2–96.5)
SSLs ≥ 10 mm	43.6 (30.3–57.7)	95.6 (93.2–97.3)	55.8 (42.6–68.3)	93.0 (91.3–94.4)	89.6 (86.6–92.2)
All SSLs	38.8 (30.1–48.1)	97.8 (97.0–98.4)	55.3 (45.7–64.5)	95.8 (95.2–96.4)	94.0 (92.8–95.0)

The 95% confidence intervals (95% CI) are given in parentheses.

NPV, negative predictive value; PPV, positive predictive value; SSL, Sessile‐serrated lesions.

Our results revealed that although 74 lesions (61.2%) did not fit the WASP classification, they were histopathologically identified as SSLs. In addition, 50 lesions (41.3%) were histopathologically diagnosed as SSLs despite lacking any of the four characteristics of WASP. The other endoscopic features commonly observed in these lesions include proximal colon location (60%), size greater than 5 cm (60%), flat shape (12%), varicose microvascular vessels (12%), and mucus cap (2%).

## Discussion

To the best of our knowledge, this is the first study evaluating the use of endoscopic characteristics and the WASP classification for the diagnosis of SSLs in the Vietnamese population. SSLs have been proven to have malignant potential and may substantially contribute to postcolonoscopy CRC.^22^ According to the third Asia‐Pacific consensus recommendations on CRC screening and postpolypectomy surveillance, SSLs are premalignant lesions that should be detected and removed.^23^ However, the prevalence of SSLs is likely underestimated in Asia,^24^ and it is crucial to perform vigilant examinations during colonoscopy, particularly in the right colon. The SSL detection rate also varies among endoscopists.^25^, ^26^, ^27^ By identifying the typical endoscopic features of SSLs, endoscopists may improve polyp management and potentially reduce the incidence and mortality of CRC.^28^ In our study, proximal location, size larger than 5 mm, flat morphology, irregular shape, varicose microvascular vessels, and dark spots inside the crypts were significant endoscopic features for the diagnosis of SSLs.

Our data revealed that most SSLs were located in the proximal colon, which was consistent with the findings of previous studies.^28^, ^29^, ^30^, ^31^ Kashida et al. indicated that SSLs were predominantly found in the proximal colon, while HPs were mostly found in the distal colon.^32^ Moreover, Hasegawa et al. examined 107 SSLs to distinguish them from other serrated lesions and reported that SSLs were preferentially located in the proximal rather than the distal colon.^33^ In clinical practice, bowel preparation tends to be poorer in the proximal colon; consequently, it is crucial to pay attention to adequate bowel preparation and carefully examine the proximal colon to avoid missing lesions.

Previous studies demonstrated that flat morphology, size >5 mm, and irregular shape were independent diagnostic factors for SSLs.^7^, ^34^, ^35^, ^36^ These findings were consistent with our results. Hazewinkel et al. demonstrated that irregular shape (OR: 3.17; 95% CI: 1.59–6.29) was an endoscopic predictor of SSL pathology.^7^ Ishigooka et al. also reported that there was a significant relationship between flat surface lesions and SSLs compared with traditional serrated adenomas and HPs (*P* < 0.001).^35^ Furthermore, Pereyra et al. examined 440 polyps, including 34 SSLs, 135 HPs, and 249 adenomas, and reported that a flat morphology (OR: 3.81, *P* = 0.002) was an independent diagnostic feature for SSLs.^28^ Therefore, endoscopists must be aware of these flat polyps to improve their detection.

According to our results, two endoscopic characteristics on NBI, varicose microvascular vessels and dark spots inside the crypt, were found to be independent features of SSLs. These findings were also reported by Hazewinkel et al. and Uraoka et al.^7^, ^31^ Uraoka et al. analyzed 89 lesions, including 38 SSLs and 41 HPs, and multivariate analysis of these lesions revealed that varicose microvascular vessels (OR: 8.2, *P* = 0.001) were significant diagnostic factors for SSLs.^31^ One theory is that SSLs alter the contour of the mucosal folds, and one‐third of polyps exhibit alterations in the underlying vascular pattern.^8^ In addition, Murakami et al. summarized the features of SSLs in their review report, indicating that SSLs with small dark spots and varicose microvascular vessels on magnifying NBI effectively discriminate SSLs from HPs.^34^

The specificity, negative predictive value, and accuracy of the WASP classification in our research were all greater than 90% for identifying SSLs. These values were even greater than those observed for SSLs smaller than 10 mm. When the WASP classification was introduced in 2016, a study was carried out to validate this classification system for the endoscopic differentiation of adenomas, HPs, and SSLs <10 mm.^12^ For this reason, we conducted a more detailed analysis to evaluate the diagnostic performance of the WASP classification for SSLs <10 mm. In this study, the accuracy of optical diagnosis for detecting SSLs was 0.74 (0.66–0.82) before training and 0.86 (0.80–0.91) after training. The findings of this study indicated the additional value of the WASP classification to existing characterization methods. However, our study differs from this study in that we not only assessed the performance of the WASP classification for SSLs smaller than 10 mm but also investigated SSLs with a size ≥10 mm.

In our study, the performance of the WASP classification was lower for SSLs ≥10 mm than for SSLs <10 mm. This may be because SSLs ≥10 mm are more likely to exhibit dysplasia.^30^ In addition, these lesions have several features, including pedunculated morphology, double elevation, central depression, and reddishness.^16^ The four SSL‐like features in the WASP classification, such as cloudy surfaces, indistinctive borders, irregular shapes, or dark spots inside the crypt, are less frequent in these lesions. Zhang et al. also indicated that dark spots were more common in SSA/Ps smaller than 10 mm.^37^

Regarding the performance of the WASP classification for diminutive SSLs (size <5 mm), Vleugels et al. reported high accuracy rates above 97% in the proximal colon and rectosigmoid.^38^ Conversely, Soons et al. reported that the diagnostic accuracy of the WASP classification for diminutive SSLs was lower, at 0.51 (95% CI: 0.46–0.56) and 0.55 (95% CI: 0.49–0.60, *P* = 0.119) before and after training, respectively.^39^ Another study was conducted with the participation of nonexpert endoscopists, and the accuracy of the WASP classification in differentiating SSLs was high, at 92% (95% CI 0.87–0.95).^40^ In Thailand, Netinatsunton et al. carried out research on the outcome of the WASP criteria in sessile polyps, and the participating endoscopists were also nonexpert endoscopists without prior training. Nonetheless, this study revealed that the WASP classification yielded false positives in diagnosing SSLs.^41^ Therefore, further research is needed to assess the diagnostic performance of the WASP classification in daily practice for both expert and nonexpert endoscopists.

In our study, while the specificity, negative predictive value, and accuracy of the WASP classification were high, the sensitivity was relatively low. As a result, we also examined lesions in which pathology revealed SSLs, but none of the four characteristics of the WASP classification were present. We found that other endoscopic features commonly observed in these lesions include the presence in the proximal colon, size greater than 5 mm, flat morphology, and varicose microvascular vessels. All four of these characteristics were significantly associated with SSLs according to our multivariate analysis. Consequently, in clinical practice, it is necessary to pay attention to these endoscopic features in addition to the WASP classification to maximize the sensitivity of diagnosing SSLs.

This study has several limitations. First, this was a single‐center study. Second, the study participants were symptomatic patients who underwent colonoscopy. Currently, there is no nationwide colorectal cancer screening program for asymptomatic individuals in Vietnam. Hence, the proportion of asymptomatic individuals examined in our hospital was significantly lower than that of patients with lower gastrointestinal symptoms. These limitations could limit the generalizability of the study findings. Third, chromoendoscopy methods, such as indigo carmine and crystal violet, are not available at our institution; therefore, we cannot evaluate pit patterns specific to SSLs, such as type II open patterns. Fourth, the data about the diagnostic ability of the WASP classification before the CATCH project in our study are unavailable.

## Conclusion

In conclusion, proximal location, a size larger than 5 mm, flat morphology, irregular shape, varicose microvascular vessels, and dark spots inside the crypts were significantly associated with SSL pathology. Additionally, the WASP classification had high accuracy in the diagnosis of SSLs, especially those less than 10 mm. Increasing the awareness of SSL‐specific features and WASP classification contributes to improving the detection rate, optimizing diagnosis, promoting complete resection of SSLs, and ultimately reducing the incidence of CRC.
